# Comprehensive Facial Injury Score and Facial Injury Severity Score as Injury Severity Indicators: A Comparative Study

**DOI:** 10.3390/jcm15124497

**Published:** 2026-06-10

**Authors:** Yunn Shy Chan, Siti Salmiah Mohd Yunus, Syed Nabil

**Affiliations:** 1 Department of Oral and Maxillofacial Surgery, Hospital Sultan Abdul Halim, Sungai Petani 08000, Kedah, Malaysia; chanys@moh.gov.my; 2 Department of Oral and Maxillofacial Surgery, Faculty of Dentistry, Universiti Kebangsaan Malaysia, Kuala Lumpur 50300, Malaysia; sitisalmiah@hctm.ukm.edu.my

**Keywords:** maxillofacial injuries, trauma severity indices, injury severity score, length of hospital stay, interdisciplinary communication

## Abstract

**Background**: The Comprehensive Facial Injury Score (CFI) and Facial Injury Severity Score (FISS) are validated scoring systems used to assess the severity of maxillofacial injuries. Direct comparative evidence of their associations with multiple clinical severity indicators remains limited. This study aimed to compare CFI and FISS in assessing the severity of maxillofacial injuries, including length of hospital stay (LOS), need for surgical intervention (NSI), overall surgical time (OST), and need for interdisciplinary management (IDM). **Methods**: A retrospective review of 280 medical records of patients with maxillofacial injuries between January 2016 and December 2021 was conducted. Pearson and point-biserial correlation analyses were performed to assess the association between CFI and FISS scores with all four outcome variables. **Results**: CFI demonstrated strong positive correlations with LOS (r = 0.65, *p* < 0.001) and OST (r = 0.74, *p* < 0.001), compared to moderate correlations for FISS (r = 0.41 and r = 0.54, respectively; both *p* < 0.001). For NSI, CFI showed a strong correlation (r_pβ_ = 0.612, *p* < 0.001), whereas FISS showed a moderate correlation (r_pβ_ = 0.373, *p* < 0.001). For IDM, CFI demonstrated a moderate correlation (r_pβ_ = 0.384, *p* < 0.001) while FISS showed only a weak correlation (r_pβ_ = 0.150, *p* = 0.012). Both scoring systems demonstrated convergent validity, confirming they measure a related underlying construct of maxillofacial injury severity. **Conclusions**: CFI demonstrated stronger correlations with all four severity indicators compared to FISS, suggesting a stronger association in this study population. These findings support the potential utility of CFI in stratifying maxillofacial injury severity.

## 1. Introduction

Facial bones are complex structures consisting of 14 paired and non-paired bones. Injuries to these bones can be described as three types: isolated fracture refers to a fracture involving only a single facial bone; complex fracture refers to a fracture involving more than one facial bone but in the same subunit of the face, and pan-facial fracture, which indicates a fracture involving more than three facial subunits [1]. Facial fractures are often associated with other injuries such as head injury, spinal injury and limb fractures, which might necessitate a lengthy hospitalisation [2,3]. Hospitalisation due to these injuries can lead to nosocomial infections, prolonging hospital stay [3]. Prolonged hospitalisation can lead to economic implications, both directly through the total cost of treatment and hospitalisation, and indirectly by impacting the patient’s ability to work and be financially productive during treatment and recovery [4,5]. In nations with a public healthcare system, longer hospitalisation periods will also impose economic cost on public expenditure [6].

There have been several facial injury assessment tools that have been used to assist in assessing its severity, such as Cooter and David Score, Facial Fracture Severity Score (FFSS), Maxillofacial Injury Severity Score (MFISS), Facial Injury Severity Scale (FISS), Complex Craniofacial Fractures model, and Comprehensive Facial Injury (CFI). The two most common tools are FISS and MFISS [5]. FISS was proposed by Bagheri et al., where it divided the face into three parts: the upper third, middle third, and lower third, and then scores were given based on the location of the facial bone fracture [7]. This scoring system also assigned a score for facial lacerations exceeding 10 cm in total length. The CFI score is a more recently introduced scale, first proposed in 2019 [8]. CFI has been shown to measure the severity of maxillofacial injuries, as it correlates with the types of treatment required [9,10]. Compared to FISS, the CFI score considers displaced or comminuted fractures, assigning higher points to them [8]. This parameter is considered important for estimating the level of care required, allowing clinicians to plan therapeutic strategies, estimate surgical and hospitalisation duration, and judge the overall outcome [11]. The CFI, however, has not been compared with the previously widely accepted FISS on management-related parameters. This study aims to compare the associations of CFI and FISS scores with management-related severity indicators, including length of hospital stay, the need for surgical intervention, overall surgical time, and the need for interdisciplinary management.

## 2. Materials and Methods

The study protocol adhered to the ethical principles outlined in the Declaration of Helsinki: Ethical Principles for Medical Research Involving Human Subjects. Ethical approval from the Research Ethics Committee, Universiti Kebangsaan Malaysia (UKM PPI/111/8/JEP-2021-735), was obtained prior to initiating the retrospective data collection. The sample comprised all new referrals to the Oral and Maxillofacial Surgery Department at Hospital Canselor Tuanku Muhriz, Universiti Kebangsaan Malaysia (HCTM), for oral and maxillofacial injuries from 1 January 2016 to 31 December 2021. The list of patients was obtained from the department’s clinical census.

A total of 280 subjects were required based on a priori sample size calculation, assuming a medium effect size (r = 0.3) following Cohen’s (1988) convention, a two-tailed significance level of α = 0.05, and a statistical power of 80% [12]. All patients during the research period were screened for inclusion and exclusion criteria. Patients who met the inclusion and exclusion criteria were assigned a number label, and a simple random sampling using computer-generated random numbers was performed to select the required sample.

The inclusion criteria were cases with complete medical records, which consisted of documented medical notes regarding the facial injury and CT scan data. The exclusion criteria were fatal cases and patients transferred to other hospitals for further management, as complete outcome data could not be ascertained beyond the point of transfer. The independent variables collected included demographic data (age, gender, ethnicity) and clinical data (source of referral, clinical presentations, diagnosis, aetiology of injuries). The dependent data were the CFI and FISS results, length of hospital stay (LOS), the need for surgical intervention for maxillofacial injuries (NSI), overall surgical time (OST), and presence of interdisciplinary management (IDM). The definitions of the dependent outcomes are described in Table 1.

For the CFI and FISS scoring process, fracture assessment was performed by reviewing the CT scan images. Soft tissue injury scoring was based on the documented clinical records. When classification was ambiguous based on either CT scan images or clinical documentation, a consensus decision was reached through discussion among the investigators. For this study, only laceration wounds were considered soft tissue injuries (STI); abrasion wounds and contusions were excluded as FISS and CFI scoring systems only score laceration wounds. We defined simple laceration as wounds less than 10 cm in size with no involvement of the surrounding vital structures. In contrast, complicated laceration was defined as wounds greater than 10 cm or when involving surrounding vital structures such as the facial nerve, the trigeminal nerve, the salivary duct, the lachrymal drainage system, loss of tissues, human or animal bite, gunshot wound, or the presence of retrobulbar haematoma. For facial bone fractures, upper face fractures include the orbital roof and the frontal sinus. Midface skeletal fractures included Le Fort fractures, naso-orbital-ethmoid fractures, zygomatico-maxillary complex fractures, nasal fractures, and orbital floor and medial orbital wall fractures. Lastly, lower face fracture refers to fracture of the mandible. In addition, dental trauma included luxation, fracture, or a combination of both.

Admission type was divided into acute or elective admission. Acute admission was defined as emergency or urgent treatment, including pain control, feeding, airway management, or early surgical intervention. Acute admissions were subdivided into admission to the emergency department (ED) immediately following trauma and acute admission to the ward following transfer from ED. Elective admission was the re-admission of patients after discharge from the previous acute admission for the purpose of undergoing surgery.

The data were analysed quantitatively using IBM SPSS Statistics version 26.0 (SPSS Inc., Chicago, IL, USA). Independent samples *T*-tests were used to compare CFI and FISS scores across outcome groups. A Pearson correlation was conducted to assess the degree of association between CFI and FISS scores, to examine whether the two scoring systems demonstrate convergent validity in measuring maxillofacial injury severity. Pearson and point-biserial correlation tests were performed to assess the association between the CFI and FISS scores with LOS, NSI, OST, and IDM. Significance level was set at *p* < 0.05 for all statistical tests. ChatGPT (OpenAI, GPT-4) and Claude Sonnet 4.6 (Anthropic) were used for superficial text editing assistance during manuscript preparation.

## 3. Results

A total of 1875 medical records of patients with maxillofacial injuries were initially screened using data from the department’s clinical census. Following screening with the inclusion/exclusion criteria, 625 records were removed from the sampling population for not meeting the inclusion/exclusion criteria. Of the remaining eligible 1250 patients, number labels were assigned, and 280 samples were randomly selected. These 280 formed the final sample for this study on which data were extracted.

This sample consisted of 223 (79.6%) males and 57 (20.4%) females. The Malay ethnic group formed the majority of the sample with 167 patients (59.6%), followed by Chinese with 68 patients (24.3%), Indian with 30 patients (10.7%) and other ethnicities with 15 patients (5.4%). The mean age of patients was 35.02 (SD ±16.02) with the youngest aged 12 and the oldest aged 85 at the time of injury. Table 2 summarises the demographic characteristics of the study population.

The most common cause of maxillofacial injuries was road traffic accidents (83.2%), followed by falls (10.4%), assaults (5.0%), sport injuries (1.1%), and the least common was industrial injuries (0.4%). Regarding the type of maxillofacial injury sustained, the most common combination was laceration STI with fracture (46.1%), followed by facial bone fractures only (30.0%), while the least common was a combination of facial bone fracture and dental trauma (2.1%). Among laceration wounds, simple lacerations accounted for 61.8%, whereas complicated lacerations accounted for 6.1%. Of all facial bone fractures sustained, the most common type of fracture involved the midfacial skeletal fractures (47.5%), followed by lower face fractures (17.5%). The least common was a combination of upper face and lower face fractures which only accounted for 0.4%. Table 2 shows the distribution of injuries of the study population.

The mean total length of hospital stays is 110.24 ± 97.49 h with range: 4–370 h. The mean length of acute admission in the ED is 5.81 ± 1.26 h (range: 3–10 h). The mean length of stay for acute admission to the ward is 119.92 ± 90.27 h (range: 17–353 h). Of 280 patients, 153 patients (54.6%) required admission to the ward from the ED following injury. A total of 124 patients (44.3%) required elective admission to the ward with a mean length of stay of 86.7 ± 25.17 h (range: 44–143 h). Table 3 shows the distribution of the length of hospital stays.

The severity of maxillofacial injuries was indicated by six parameters, which were the FISS score, CFI score, LOS, NSI, OST, and IDM (Table 4). The mean FISS score was 2.36 ± 2.29 (range: 0–18), while the mean CFI score was 7.87 ± 5.39 (range: 1–27.5). The mean total LOS is 110.24 ± 97.49 h (range: 4–370 h). Of the 280 cases, 151 (53.9%) underwent ORIF for maxillofacial fractures, whereas no surgical intervention was needed in 129 cases (46.1%). Among the 151 patients who underwent ORIF, the mean overall surgical time (OST) was 188.32 ± 86.52 min (range: 45–420 min). A total of 204 patients (72.9%) sustained combined maxillofacial and other types of injury, such as head injury, orthopaedic injury, thoracic injury, abdominal injury, and laryngeal injury, requiring co-management with other specialties including neurosurgery, orthopaedics, general surgery, otolaryngology, and ophthalmology. Only 76 patients (27.1%) who sustained facial injuries had no IDM.

Of the 204 patients who had IDM, half were co-managed by OMFS with another specialty (Table 5). Forty-nine (24.0%) of these cases were co-managed with ophthalmology, 23 patients (11.3%) with orthopaedics, 19 patients (9.3%) with neurosurgery, eight patients (3.9%) with general surgery, and three patients (1.5%) with otorhinolaryngology (ENT). The remaining 102 patients (50.0%) were co-managed by three or more specialties, including OMFS. Across all 280 patients, ophthalmology was the most frequently involved specialty, attending to 141 patients (69.1%). This was followed by neurosurgery with 90 patients (44.1%), orthopaedics with 80 patients (39.2%), general surgery with 33 patients (16.2%), and the least was otorhinolaryngology with five patients (2.5%).

A Pearson correlation was conducted to determine the relationship between CFI and FISS scores. CFI and FISS demonstrated a very strong positive correlation, r(278) = 0.736, *p* < 0.001, supporting convergent validity between the two scoring systems. A Pearson correlation was then conducted to determine the relationship between CFI and FISS scores with LOS. The variables CFI score and LOS were strongly positively correlated, r(278) = 0.647, *p* < 0.001, while FISS score and LOS were moderately positively correlated, r(278) = 0.410, *p* < 0.001. For the relationship between CFI scores and FISS score with overall surgical time, the variables CFI score and OST were very strongly positively correlated, r(278) = 0.737, *p* < 0.001. In contrast, FISS score and OST were moderately positively correlated, r(278) = 0.539, *p* < 0.001.

A point-biserial correlation was conducted to determine the relationship between CFI and FISS scores with the NSI. There was a strong positive correlation between CFI score and the NSI, which was statistically significant (r_pβ_ = 0.612, *n* = 280, *p* < 0.001), compared to a moderate positive correlation between FISS score and the NSI (r_pβ_ = 0.373, *n* = 280, *p* < 0.001). For the relationship between CFI and FISS scores with the IDM, there was a moderate positive correlation between CFI score and IDM, which was statistically significant (r_pβ_ = 0.384, *n* = 280, *p* < 0.001). In contrast, there was a weak positive correlation between FISS score and IDM (r_pβ_ = 0.150, *n* = 280, *p* = 0.012) (Table 6).

## 4. Discussion

The incidence of maxillofacial trauma varies across geographic regions, largely due to the differences in socioeconomic conditions [13]. This contributes to the variation in the affected populations and the aetiologies of trauma observed. The demographic findings in this study, regarding age and gender, mirror those from previous studies in this region [14,15,16]. Furthermore, the finding that road traffic accidents are the most common aetiology of maxillofacial injuries concurs with previous local reports [14,17]. One notable difference is that most previous studies found STI to be the most common type of facial injury [14,18]. However, our study reported that 32% of cases did not sustain STIs. This result is likely because our study included only laceration wounds as STIs, while contusions and abrasions were not considered. This explains the relatively high proportion of cases with “facial fracture only,” as these patients may have sustained fractures with overlying contusions or abrasions. As both CFI and FISS assign points exclusively to laceration wounds, this may result in an underestimation of the overall soft-tissue injury burden, an acknowledged inherent limitation of both scoring systems. Regarding the pattern of facial bone fractures, the predominance of midface fractures is consistent with most previous studies [14,19].

FISS was introduced in 2006 to quantify and reliably predict the severity of maxillofacial injuries by estimating the economic cost required for surgical treatment [7]. It was described as the best available tool for assessing the severity of maxillofacial trauma at that time [5,20]. The severity of facial injuries as scored by FISS has been found to correlate with surgical charges for maxillofacial injury treatment and with hospital length of stay [7]. Additionally, FISS has been shown to be associated with the need for surgical intervention and interdisciplinary management [21]. The CFI score, introduced later, aimed to improve upon the FISS by using surgical time instead of a non-standard, cost-related outcome and by being more specific in classifying the fracture severity, including the description of displaced fractures [8]. The CFI score has been found to correlate with surgical time and length of hospital stay [10,11]. Higher CFI scores have also been suggested as predictors of associated neurological injuries [10]. This study considered all previously reported severity indicators associated with either or both indices (LOS, NSI, OST, and IDM) and directly compared the association across these indicators.

The LOS is commonly used as an indirect indicator of injury severity in trauma research, including maxillofacial and craniofacial injuries. Longer LOS typically indicates greater injury complexity, the need for surgical intervention, institutional factors such as bed availability and discharge policies, and patient-related variables including comorbidities, age, and socioeconomic background. This means that LOS is not a specific indicator of maxillofacial injury severity per se, but rather reflects the patient’s overall therapeutic needs and should therefore be interpreted with caution. Despite this, LOS is one of the severity indicators found to be correlated with both FISS and CFI scores [7,10,11]. In this study, when comparisons were made, we found that the CFI score demonstrated a stronger correlation with LOS compared to the FISS score. Rawat et al. reported an increasing trend in LOS in high dependency units (HDU) with increasing CFI cluster number, up to a CFI score of 25 [10]. It also reported that the duration of HDU stay was statistically significant among the clusters (*p* < 0.001) [10]. Aita et al. meanwhile reported that the patients with FISS scores greater than 3 had 18 times the likelihood of being treated for more than three days [21].

More severe injuries typically require longer operative times, either because of multiple fracture sites or the increased complexity of the surgical procedure. Surgical time can also be influenced by other factors, such as the operating surgeon experience, the availability of advanced equipment, and intraoperative complications. However, surgical time remains a more specific indicator of maxillofacial injury severity compared to LOS, particularly as, in this study, OST was considered only for the facial trauma procedures performed on each patient. This study found that the CFI score showed a stronger correlation with OST than the FISS score. Rawat et al. showed that the total surgical time was statistically significant across all the CFI score clusters (*p* < 0.001) [10]. Canzi et al. reported that the CFI score achieved 88.7% accuracy in predicting whether the surgical time for specific injuries would exceed a defined threshold, and 90.2% accuracy in predicting the maximum surgical time overall [8].

Patients with more complex or displaced fractures, or those with functional impairment, are more likely to require surgical intervention and therefore could indicate a higher level of trauma severity. The decision to proceed with surgical intervention may also be influenced by institutional factors, surgeon preference, patient comorbidities, or resource availability. This study found that both the FISS and CFI scores demonstrated statistically significant correlations with the NSI. The CFI score showed a stronger correlation than the FISS, as expected, given that the CFI considers fracture severity, specifically fracture displacement, as one of its parameters. In both indices, higher scores were associated with a greater likelihood of surgical intervention. This finding is consistent with previous reports. Rawat et al. found that the cases managed conservatively had a median CFI score of 1 (range: 0–9), while the cases managed surgically had a median CFI score of 7 (range: 1–31) [10]. For the FISS scale, Aita et al. reported that the patients with a FISS score greater than 5 had 18 times the likelihood of requiring surgical intervention [21].

In general, multidisciplinary management of a trauma patient reflects the extent and systemic impact of the injuries rather than localised facial trauma alone. It is therefore more closely aligned with the LOS outcome, in which non-maxillofacial injuries contribute to the need for multidisciplinary care. In this study, ophthalmology was the most commonly involved co-managing specialty. This is consistent with the sample in this study, which showed that the majority sustained midfacial fractures that frequently involve the orbital walls. Neurosurgical involvement, which is the second most common, is expected as intracranial injury is a well-known concomitant of maxillofacial trauma [17]. Involvement of specialties managing distant non-head and neck injuries, such as orthopaedic or general surgery, is also expected, given that the majority of the patients in this cohort sustained injuries from road traffic accidents, predominantly involving motorcyclists, who are particularly vulnerable to multisystem injuries. Thoren et al. reported that 25.2% of the patients with maxillofacial injuries also sustained concomitant injuries in other parts of the body requiring support from other specialities, with orthopaedics and neurosurgery being most frequently involved [22]. As with other indicators, both the FISS and CFI scores showed significant correlation with the need for IDM, with the CFI score demonstrating a stronger correlation. The study by Hwang et al. showed that the two most common complications suffered by patients with panfacial fractures were neurological and ophthalmic complications [23]. Moreover, the five mostly co-referred specialties reported by Hwang et al. were neurosurgery, orthopaedic surgery, ophthalmology, otolaryngology, and thoracic surgery [23], which is consistent with the findings of the present study. Rawat et al. demonstrated a significant association between the CFI score and the prevalence of head injury [10]. For the FISS score, Aita et al. stated that the patients with maxillofacial injuries with a FISS score greater than 5 had a 6.6 times higher likelihood of requiring support from other specialties (*p* < 0.0001) [21]. Another study by Siregar et al. found a significant relationship between FISS score and involvement with other specialties when FISS was greater than 3 (*p* = 0.044), with neurosurgery as the most common specialty [24]. Additionally, panfacial fracture patients with FISS greater than 11 had a significant chance of requiring multi-professional treatment [1].

A Pearson correlation was conducted to examine the degree of association between the CFI and FISS scores across the study sample. The two scoring systems were found to be very strongly correlated with one another, suggesting convergent validity, meaning that both indices appear to measure a related underlying construct of maxillofacial injury severity. This is clinically expected, as both CFI and FISS are derived from similar anatomical regions and injury types and incorporate both soft-tissue and skeletal injury components into their scoring. However, despite this shared conceptual basis, CFI consistently showed stronger correlations with all four outcome variables than FISS. This suggests that, while the two indices converge in their overall measurement of severity, CFI shows stronger associations with clinically meaningful management-related indicators, such as surgical management and length of stay. The stronger correlations observed with CFI may be attributed to its more granular classification of fracture severity and its high descriptive capacity, which supports more precise patient differentiation than the predominantly anatomically oriented FISS [8,25]. This refinement may contribute to the stronger associations observed between CFI and outcomes reflecting surgical complexity and resource utilisation. The simplicity and ease of use of each scoring index are other factors that were not assessed in this study. However, FISS, a simpler scoring system based on a point-based summation of anatomical fractures, may have an advantage for clinical adoption and could influence uptake among clinicians and administrators [25,26]. We suggest that CFI may be more appropriately used by OMFS surgeons for more detailed assessment, surgical planning, and resource allocation following referral. In contrast, FISS offers a simpler anatomy-based point summation and may be more readily applied during initial assessment, including in emergency or resource-limited settings, where rapid estimation of facial injury severity is required.

Several limitations should be considered when interpreting the findings of this study. First, the retrospective design introduces the possibility of incomplete or inconsistent documentation in medical records, potentially affecting the accuracy of data extraction. Furthermore, CFI and FISS scoring in this study was performed by a single assessor without formal blinding to clinical outcomes. Although the scoring is based largely on objective clinical and radiographic findings, formal blinding and independent dual scoring would have strengthened the reliability and reproducibility of the data. Second, as a single-centre study, the findings may not be fully generalisable to other institutions or regions with differing patient demographics, trauma mechanisms, or healthcare infrastructure. The exclusion of cases with fatal outcomes or those transferred out may also result in the under-representation of these patient subgroups, which could similarly limit generalisability. Future multicentre prospective studies across diverse trauma populations are therefore recommended, as the inclusion of a more heterogeneous, larger sample would enable broader generalisability and further validation of the findings of this study. Finally, potential confounders such as age, mechanism of injury, polytrauma, comorbidities, surgeon experience, and institutional logistics, including hospital bed availability, were not adjusted for and may have influenced the observed associations between injury severity scores and management-related indicators. Notwithstanding these limitations, this study has several notable strengths. It is one of the few studies to directly compare the CFI and FISS scoring systems with respect to multiple clinically relevant management-related severity indicators. The use of four distinct outcome variables, namely LOS, NSI, OST, and IDM, provided a comprehensive assessment of injury severity rather than relying on a single proxy measure. These findings contribute meaningful evidence to the growing body of literature on maxillofacial trauma scoring systems.

## 5. Conclusions

Overall, this study found that the CFI score more closely reflected the severity of maxillofacial injuries compared to the FISS score, demonstrating stronger correlations with all four assessed parameters: LOS, NSI, OST, and IDM. Both scoring systems demonstrated convergent validity, indicating that they measure a related construct of maxillofacial injury severity. However, CFI demonstrated stronger associations with these clinically relevant, management-related severity indicators.

## Figures and Tables

**Table 1:** Definitions of the dependent outcome variables.

Variables	Definitions
Length of hospital stay (LOS)	Total duration from hospital admission to discharge, recorded in hours. Where applicable, both acute admission and subsequent elective admission related to definitive management of the same injury episode were included.
Need for surgical intervention (NSI)	Requirement for operative management of facial bone fractures, specifically open reduction and internal fixation (ORIF).
Overall surgical time (OST)	Duration of definitive surgical intervention for facial traumatic injuries, recorded in minutes. This excluded anaesthesia induction, recovery time, and procedures performed for non-facial injuries.
Interdisciplinary management (IDM)	Involvement of other medical or surgical specialties for concomitant injuries associated with maxillofacial trauma.

**Table 2:** Demographic and clinical data of the study population.

Variable	Category	*n* (%)
**Age**		35.02 (±16.02) *
**Gender**	Male	223 (79.6)
	Female	57 (20.4)
**Ethnicity**	Malay	167 (59.6)
	Chinese	68 (24.3)
	Indian	30 (10.7)
	Others	15 (5.4)
**Aetiology of maxillofacial injuries**	Road traffic accident	233 (83.2)
	Fall	29 (10.4)
	Assault	14 (5.0)
	Sport injury	3 (1.1)
	Industrial injury	1 (0.4)
**Types of maxillofacial injuries**	Laceration STI only	15 (5.4)
	Facial fractures only	84 (30.0)
	Combination of laceration STI and fractures	129 (46.1)
	Combination of laceration STI and dental trauma	10 (3.6)
	Combination of fractures and dental trauma	6 (2.1)
	Combination of laceration STI, fractures and dental trauma	36 (12.9)
**Laceration severity**	Simple laceration	173 (61.8)
	Complicated laceration	17 (6.1)
	No laceration (abrasion and contusion only)	90 (32.1)
**Types of facial bone fractures**	Upper face	4 (1.4)
	Midface	133 (47.5)
	Lower face	49 (17.5)
	Upper and midface	30 (10.7)
	Upper and lower face	1 (0.4)
	Midface and lower	35 (12.5)
	Upper face, midface, and lower face	3 (1.1)
	No fracture	25 (8.9)
**Treatments for injuries**	ORIF for facial bone fractures	151 (53.9)
	Closed reduction for facial bone fractures	104 (37.1)
	No facial bone fracture	25 (8.9)

* Mean (SD); STI: soft tissue injury; ORIF: open reduction and internal fixation surgery.

**Table 3:** Distribution of length of hospital stays (hours).

Variable	Category	*n* (%)
Total length of hospital stays *		110.24 (97.49), min: 4, max: 370
Length of acute admission in ED *		5.81 (1.26), min: 3, max: 10
Length of acute admission in ward *		119.92 (90.27), min: 17, max: 353
Length of elective admission in ward *		86.7 (25.17), min: 44, max: 143
Need acute admission in ward	Yes	153 (54.6)
	No	127 (45.4)
Need elective admission in ward	Yes	124 (44.3)
	No	156 (55.7)

* Mean (SD); ED: emergency department.

**Table 4:** Distribution of severity of maxillofacial injuries.

Variable	Category	*n* (%)
FISS score *	Mean	2.36 (2.29), min: 0, max: 18
CFI score *	Mean	7.87 (5.39), min: 1, max: 27.5
LOS *	Mean	110.24 (97.49), min: 4, max: 370
NSI	Yes	151 (53.9)
	No	129 (46.1)
OST *	Mean	188.32 (86.52), min: 45, max: 420
IDM	Yes	204 (72.9)
	No	76 (27.1)

* Mean (SD); LOS: length of hospital stay; NSI: need for surgical intervention; OST: overall surgical time; IDM: presence of interdisciplinary management.

**Table 5:** Distribution of interdisciplinary management for facial injuries.

Variable	Category	*n* (%)
Interdisciplinary Pairing	OMFS + neurosurgery	19 (9.3)
	OMFS + orthopaedics	23 (11.3)
	OMFS + general surgery	8 (3.9)
	OMFS + opthalmology	49 (24.0)
	OMFS + otolaryngology	3 (1.5)
	Combination of more than two	102 (50.0)
Specialty Frequency *	Neurosurgery	90 (44.1)
	Orthopaedics	80 (39.2)
	General surgery	33 (16.2)
	Opthalmology	141 (69.1)
	Otolaryngology	5 (2.5)

OMFS: Oral and Maxillofacial Surgery; * percentages for specialty frequency sum to greater than 100%, as individual patients may have been co-managed by more than one specialty.

**Table 6:** Correlation between measures.

Measure	CFI	FISS
LOS	0.647 a,*	0.410 a,*
OST	0.737 a,*	0.539 a,*
NSI	0.612 b,*	0.373 b,*
IDM	0.384 b,*	0.150 b,**

Note: a = Pearson correlation; b = point-biserial correlation; * *p* < 0.01, ** *p* < 0.05. Correlation strength: weak (r = 0.10–0.29), moderate (r = 0.30–0.49), strong (r = 0.50–0.69), and very strong (r ≥ 0.70). LOS: length of hospital stay; NSI: need for surgical intervention; OST: overall surgical time; IDM: presence of interdisciplinary management.

## Data Availability

The data presented in this study are available on request from the corresponding author due to privacy restrictions.

## Abbreviations

The following abbreviations are used in this manuscript:

CFI	Comprehensive Facial Injury score
FISS	Facial Injury Severity Score
LOS	Length of hospital stay
NSI	Need for surgical intervention
OST	Overall surgical time
IDM	Interdisciplinary management
ORIF	Open reduction and internal fixation
OMFS	Oral and Maxillofacial Surgery
HCTM	Hospital Canselor Tuanku Muhriz
STI	Soft tissue injury
ED	Emergency department
HDU	High dependency unit
GA	General anaesthesia
FFSS	Facial Fracture Severity Score
MFISS	Maxillofacial Injury Severity Score
UKM	Universiti Kebangsaan Malaysia
ENT	Otorhinolaryngology
SD	Standard deviation
IBM	International Business Machines
SPSS	Statistical Package for the Social Sciences
